# Flower colour polymorphism in *Anemone coronaria* correlates with the activity pattern and colour preferences of its visitors

**DOI:** 10.1093/aobpla/plag009

**Published:** 2026-02-18

**Authors:** Tzlil Labin, Banisha Phukela, Casper J van der Kooi, Tamar Keasar, Yuval Sapir

**Affiliations:** Department of Evolutionary & Environmental Biology, University of Haifa, 199 Aba Khoushy Ave., Mount Carmel, Haifa 3498838, Israel; Yehuda Naftali Botanic Garden, School of Plant Sciences & Food Security, Tel-Aviv University, 12 Klausner Street, Tel Aviv 6997801, Israel; Groningen Institute for Evolutionary Life Sciences, University of Groningen, P.O. Box 11103, 9700 CC Groningen, The Netherlands; Department of Biology & The Environment, University of Haifa—Oranim, Tiv'on 36006, Israel; Yehuda Naftali Botanic Garden, School of Plant Sciences & Food Security, Tel-Aviv University, 12 Klausner Street, Tel Aviv 6997801, Israel

**Keywords:** *Anemone coronaria*, coleoptera, diptera, flower colour polymorphism, glaphyrid beetles, hymenoptera, pollination, poppy anemone

## Abstract

Shifts in pollinator composition and associated colour preferences can support flower colour polymorphism (FCP) across environmental gradients and along flowering seasons. We explored the geographical and seasonal turnover of insect visitors in *Anemone coronaria*, a geophyte that flowers mainly in red, purple, and white. In Israel, southern populations have red flowers whereas some northern populations are colour polymorphic. Southern populations bloom later than northern ones, and red flowers appear later than non-red flowers. We predicted corresponding changes in colour preferences of *A. coronaria*’s pollinators, from non-red in northern sites and early in the season to red in southern sites and in late season. We created experimental arrays of red, purple, and white *A. coronaria* flowers in three field sites (northern, central, and southern regions of the species’ distribution) and three time-points (early, mid, and late season) over 2 years. We recorded flower colour choices of insect visitors. Insect colour preferences varied among locations and time points. Bees and flies visited the flower arrays primarily during early season in the northern and central sites, where bees preferred purple flowers. Beetles visited the flowers mostly during late season in the southern site and preferred red flowers there. As predicted, *A. coronaria*’s purple flowers received more early-season visits in the north, and red flowers received more late-season visits in the south. These spatio-temporal trends are consistent with the species’ FCP pattern. *A. coronaria*’s broad pollinator fauna may enhance reproductive success across the plant’s wide geographical distribution and long flowering season.

## Teaser

Flower colour polymorphism is widespread, yet its ecological drivers are rarely tested across both space and time. Using experimental flower arrays of Anemone coronaria, the poppy anemone, we show that pollinator communities and their colour preferences shift predictably along a latitudinal gradient and through the flowering season. These spatiotemporal changes align with the distribution of red, purple and white flower morphs, supporting a role for pollinators in maintaining colour diversity.

## Introduction

Flower colour polymorphism (FCP) is a conspicuous manifestation of intraspecific phenotypic variation in plants. Such within-species colour variation occurs among plant populations and also, more rarely, within populations. Floral colour attracts pollinators that vary in their visual capabilities. Hence, flowering in a variety of colours is often viewed as an adaptation supporting interactions with multiple pollinator taxa (Barragán-Fonseca *et al*. 2020, Sapir *et al*. 2021, Wenzell *et al.* 2023). The evolution of insect colour vision preceded the radiation of flowering plants, suggesting that flower colours have evolved to match their pollinators’ visual capabilities rather than *vice versa* (Chittka and Menzel 1992, Chittka 1996, Vorobyev and Menzel 1999, van der Kooi and Ollerton 2020). Therefore, it is likely that plants’ interactions with diverse pollinators drive natural selection processes leading to FCP (Waser and Price 1981, Menzel and Shmida 1993, Campbell *et al*. 1997, Schiestl and Johnson 2013, Sapir *et al*. 2021). These ecological interactions can act on underlying genetic variation; in some cases, FCP reflects heritable differences in a single pigment-coding gene that can be shaped by pollinator-mediated selection (Bradshaw and Schemske 2003, Hoballah *et al.* 2007, Kellenberger *et al*. 2019).

Whereas directional selection on floral colour reduces colour variation within species, disruptive selection increases variation. Several potential biotic selective agents can maintain FCP. For instance, plants that interact with multiple pollinator taxa, each showing preference for a different colour, are expected to benefit from FCP (Jones and Reithel 2001, Sapir *et al*. 2021). Another example involves pollinators that utilize flowers of dark and light centres differently, for feeding and mating, respectively (Lebel *et al*. 2018). Non-pollinator biotic agents of selection also drive flower colour evolution (Arista *et al.* 2013, Mtileni *et al.* 2024), and contribute to FCP. Well-documented examples include interactions between pollinators and herbivores, where both mutualists and antagonists prefer the same colour morph (Irwin *et al*. 2003, Frey 2004, de Jager and Ellis 2014, Saabna *et al*. 2025). In the context of interactions with pollinators, it is important to evaluate flower colours through the colour vision systems of their visitors. Pollinators vary in their abilities to perceive and learn visual features (Peitsch *et al*. 1992, van der Kooi *et al*. 2019). The underlying physiological mechanism involves differences in photoreceptor sensitivities between common flower-visiting insect taxa, such as bees, flies, beetles, and hawkmoths (van der Kooi *et al.* 2021).

Further, FCP across a plant species’ distribution range can reflect adaptations to abiotic environmental conditions, because specific floral pigments provide defense from stressors such as drought, frost, and UV radiation (Schemske and Bierzychudek 2001, Gould 2004, Strauss and Whittall 2006, Dick *et al*. 2011, Arista *et al*. 2013, Berardi *et al*. 2013, Landi *et al*. 2015, Ortiz *et al.* 2015, Vaidya *et al*. 2018, Tenhumberg *et al*. 2023). This may select for stronger floral pigmentation in stressful environments than under benign conditions, resulting in FCP across a plant species’ distribution range (Dick *et al.* 2011, Streinzer *et al*. 2019, Dafni *et al.* 2020). Other case studies document how combined environmental factors and pollinator behaviour may lead to colour adaptations in specific habitats (Streisfeld and Kohn 2007). Finally, FCP may also result from neutral or random processes, such as drift or gene flow (Wang *et al*. 2016).

Taking the plants’ point of view, FCP can benefit plants with a wide geographical distribution through encounters with different pollinator communities in different parts of their distribution range (Sapir *et al.* 2021, Wenzell *et al*. 2023). This seems to be case, for example, for the colour-polymorphic daisy, *Gerbera aurantiaca*. Different species assemblages of its Hopliine beetle pollinators show strong preferences for specific floral colours in a geographic context (von Witt 2019, Johnson *et al*. 2020). Selection for FCP could also operate in plants that have a long flowering period, encountering different pollinators during different parts of the season (Keasar and Labin 2025). Finally, FCP can be favoured when phenological asynchrony between plants and pollinators arises. Asynchrony may occur when the environmental cues that induce flowering (e.g. day length) differ from the cues that stimulate pollinator activity (e.g. temperature) (Bartomeus *et al*. 2013). This could enable generalist plants to interact with different pollinator taxa (varying in colour preferences) across sites and years, enhancing the benefits of FCP (Maglianesi *et al*. 2020, Keasar and Labin 2025).

The focal plant of the present study, *A. coronaria* (Ranunculaceae), has a generalized pollination system, a broad distribution, and a long blooming season (Horovitz *et al*. 1975; see below). Hence, *A. coronaria* flowers encounter a diverse set of pollinator taxa, possibly contributing to the species’ FCP. We focus on Hymenoptera, Diptera, and Coleoptera as visitors of this species, and ask whether the visitors’ floral colour preferences match the plant’s colour variation pattern in time and space. Our wider research programme aims to understand the multifold selective pressures that drive FCP in *A. coronaria*. The roles of herbivores and of abiotic conditions in shaping this FCP are described elsewhere (Labin *et al*. 2025, Saabna *et al*. 2025). Here, we specifically examine pollinators as potential selective agents. We test whether the spatio-temporal variation of FCP in *A. coronaria* corresponds to variations in pollinator composition and pollinator colour preferences.

**Figure 1:**
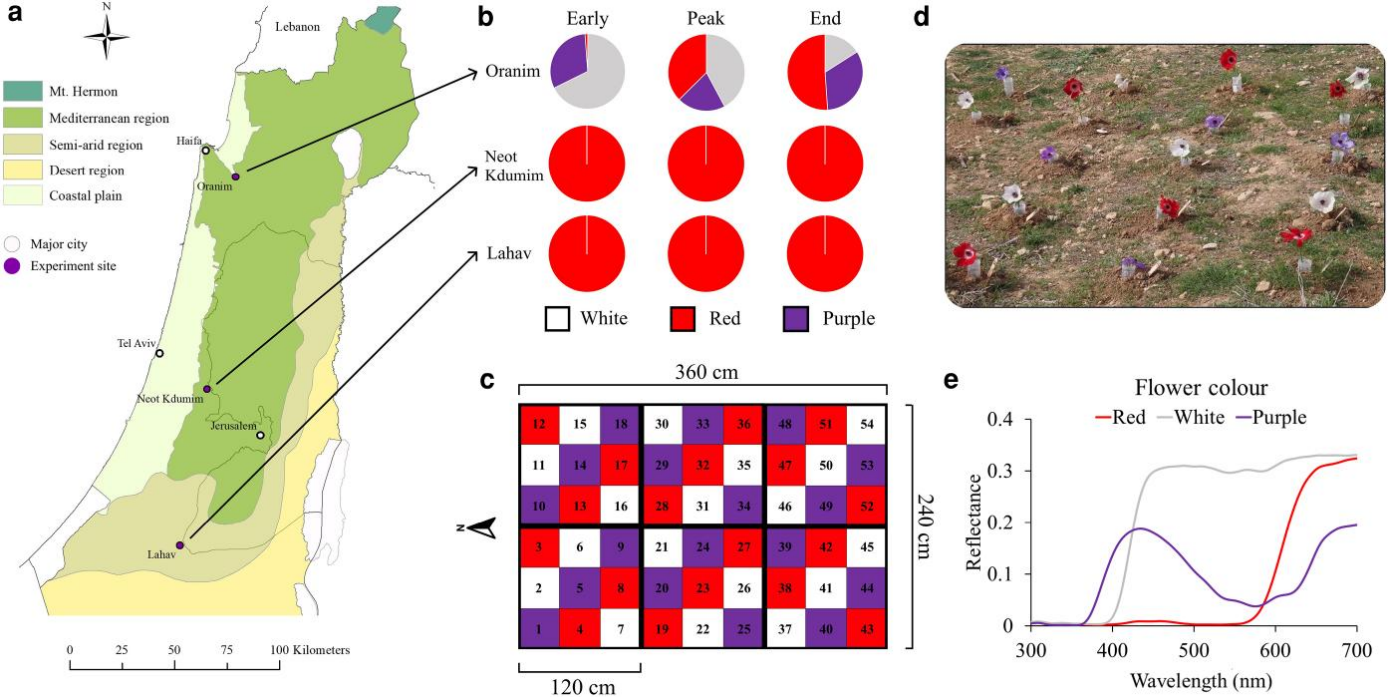
**(a)** Map of experiment sites (purple circles). Main cities are marked, for orientation, by white circles. Data for the map are based on IMS 2024, Zohary 1966. **(b)** Flower colour composition along the season is represented in pie charts (sum of two 10 × 10 m plots, averaged over 2 years), describing the nearest monitored natural anemone population to each experimental site. **(c)** Design of the flower arrays: plot matrix arrangement—colours represent the flower colour morphs of *Anemone coronaria* and numbers stand for flower positions in the plot. **(d)** Photo of the plot in the southern site. The anemones were placed in number-marked and water-filled 50 cc test tubes. **(e)** Mean reflectance spectra of each of the floral colours—red, white, and purple.

*A. coronaria* is a Mediterranean geophyte, commonly growing in grazed and open grasslands, that flowers primarily in red, purple, and white. Additional colour variants exist but are less common (Horovitz *et al*. 1975, Perevolotsky *et al*. 2011). The species has been under cultivation as an ornamental plant, which led to establishment of the genetic basis for its floral colour determination (Horovitz *et al*. 1975, Laura and Allavena 2007). *A. coronaria* is broadly distributed in Israel and neighbouring countries along a north−south rainfall gradient, from the mesic Mediterranean area in the north to the southern dry arid region (Horovitz 1991, Keasar *et al*. 2008, Dafni *et al*. 2020). The species flowers for an extended period of ca. 5 months throughout its natural range (Horovitz *et al*. 1975, Labin *et al*. 2025). An individual flower blooms for ∼2–3 weeks and is largely outcrossed and protogynous. The short female phase (2–4 days) does not overlap with the longer, subsequent male phase, and this possibly reduces self-pollination. *A. coronaria* has been observed to rely on both anemophily and on zoophily. Its only reward for flower visitors is pollen (Dafni *et al*. 2020). Within-population FCP in *A. coronaria* is spatially and temporally structured. Southern populations in Israel flower in red. Some populations in the Mediterranean areas (centre and north) are colour-polymorphic, with colour morph frequencies that vary widely between sites (Labin *et al*. 2025) and across time (Fig. 1a and b). Other populations in the Mediterranean areas flower entirely in red. The plant’s temporal flowering dynamics are also associated with FCP: northern and central populations start blooming earlier than southern ones (∼December−January versus ∼January−February, respectively). Purple and white flowers within colour-polymorphic populations bloom earlier than red ones (Labin *et al*. 2025). These spatial and temporal trends in the distribution of floral colour morphs were proposed to increase *A. coronaria’s* potential encounters with diverse pollinator assemblages (Horovitz *et al*. 1975, Dafni *et al*. 2020, Keasar and Labin 2025), and to reflect adaptation of the red morph to xeric conditions (Labin *et al*. 2025).

We examined the interactions of *A. coronaria*’s most common floral visitors (Hymenoptera, Coleoptera, and Diptera) with the plant’s different flower-colour morphs in nine combinations of seasonal timing and latitude over two flowering seasons. Previous work on the association between floral visitors and FCP in *A. coronaria* has provided observational insights on a smaller spatial and temporal scale (Horovitz 1991, Keasar *et al*. 2008, Dafni *et al*. 2020). Building on these foundations, we employed standardized and replicated flower-colour choice assays to examine visitor preferences more systematically. In addition to observations on flowers, we used pan trapping during the second year to monitor seasonal changes in the local pollinator communities. Our protocol improves previous approaches by enabling direct comparisons between turnover in the pan-trapped pollinator assemblage and that of the floral visitors of *A. coronaria*. Specifically, we interpreted seasonal changes in insect composition as reflecting the insects’ phenology if they occurred both in the flowers and in the pan traps. Seasonal changes in insect composition that occur only on the flowers are more likely to reflect changes in foraging preferences. We asked how visitor activity, composition, and floral colour choices vary along the plant’s north−south distribution range, which corresponds to a climatic gradient, and over the season. We expected the observed variation in visitor composition and colour choices to be consistent with the plant’s natural FCP pattern. That is, we predicted more pollinator visits to red *A. coronaria* in the southern part of its distribution and at the end of the flowering season than in the northern part of the plant’s distribution and in early season.

## Materials and methods

### Experimental sites

The experiment was conducted during the peak blooming seasons of *A. coronaria* (January−March) in 2022 and 2023. We replicated the experiment in the north, centre, and south of Israel, in sites that are characterized by 600, 520, and 300 mm annual rainfall, respectively (IMS 2024, Fig. 1a). These sites are located along the north−south precipitation cline that we previously surveyed to identify abiotic correlates of *A. coronaria*’s flower colour (Labin *et al*. 2025). At each site, an open space amidst local vegetation with no commercial honeybee hives in the immediate surrounding was selected as an experimental plot.

### Flower arrays

#### Experimental design

Each experimental plot (3.6 × 2.4 m) consisted of an array of 54 anemones, 18 of each colour morph, with distances of 45 cm between neighbouring flowers. The flowers were placed in 50-ml water-filled plastic test tubes, which were buried in the soil so that the rims of the tubes were at ground level. A single flower was placed per tube before each day of the experiment. In previous years, natural flower densities were recorded at the peak of the flowering season (Keasar and Sapir, unpublished data), in one northern (32.59127°N, 35.23315°E), one central (31.82413°N, 34.94359°E), and one southern population (31.42318°N, 34.51083°E). Flowers were counted in 10 1 × 1 m quadrats per site. These samples yielded an average flower density of 5.6 per m^2^, and this density was approximated in the experimental arrays. Flowers of each colour morph neighboured the two other colours (following Karron and Mitchell 2012). Flower colours were balanced across the array’s rows and columns, and between border and centre positions (Fig. 1c). The experiment was repeated three times in each site, throughout the blooming seasons of 2022 and 2023, on warm, clear, and windless days. Each monitoring session was conducted between 8.00 and 14.00 hours. Each repetition of the experiment (early—January, mid—February and late season—March), was completed within ca. 1 week across all three sites. The flowers were collected 1–2 days before a repetition, from various natural populations, and were used across all 3 sites. They were kept refrigerated in water vases between experiments, and replaced when wilted. The order of the experimental sites was altered between repetitions (early, mid, and late season) and years (2022 and 2023), starting/ending with a different site at each timepoint. The first monitored site was always exposed to freshly picked flowers. Abiotic conditions were recorded shortly before each monitoring session and are reported in Supplementary Table S1.

#### Flower colour measurements

To compare the colours of flowers and pan traps, we measured their reflectance spectra. Spectral reflectance was measured for three flowers for each colour morph, using an integrating sphere with a measurement area of circa 5 mm (technical details in van der Kooi *et al.* 2016, Stavenga and van der Kooi 2016, Fig. 1e). Reflectance spectra were recorded for the traps (see Supporting Information Supplementary Figure S1) to verify that their colours resembled the flowers (Fig. 1d and e).

#### Insect visitation recording

Each insect observed landing on an anemone flower in the plot was identified to order level, and to a more detailed taxonomic level when possible (e.g. distinguishing between honeybees and wild bees). Insects visiting flowers in the plots were collected into tubes twice a day, at 10.00 and 14.00 hour of each observation day, and refrigerated until further identification in the laboratory. As the pollination efficacy of the different floral visitors is unknown, we recorded all insects observed. We photographed the insects and recorded the numbers of red, white, and purple anemones they visited. Two observation protocols were alternated along a monitoring day: (i) Twenty minutes of recording all insects present on the flowers, while slowly walking along a fixed trail between the flowers. This protocol allowed us to detect small and motionless insects on flowers, and (2) 30 minutes of tracking of individual visitors. Each individual was followed from its first visit to a flower in the array until it left the array. This protocol enabled the recording of visit sequences.

### Pan traps

#### Experimental design

During the blooming season of 2023 (January−March), water-filled pan traps were set up on each monitoring day, about 50 m away from the experimental arrays. Thirty bowls (10 red, 10 white, and 10 purple) were placed at 10.00 hour and collected at 14.00 hour. The traps were placed in three rows separated by colour, at a distance of 1 m from each other. The trapped insects were collected at the end of each monitoring day and were later sorted and identified to family level. We then assessed whether the seasonal changes in insect activity in the flower arrays mirrored changes in the pan-trap captures (i.e. the seasonal turnover in the insects’ community composition).

### Data analysis

#### Insect visits—comparisons across time and space

We used three *χ*^2^ tests to check the insect preferences across time, space, and colour. For this, we calculated the proportion of total insect visits observed in each site (south/centre/north), repetition along the season (early/mid/late), and colour (red/white/purple). We conducted a *χ*^2^ test for goodness of fit to compare the number of insect visits during each repetition to random expectation (namely, that each insect order contributes one third of the total visits observed per site). We used similar tests to compare insect visits across sites, and the proportion of insect visits to each flower-colour during each repetition (early/mid/late), to the randomly expected uniform distribution.

#### Insect composition—comparing flowers and traps

We used non-metric multidimensional scaling (NMDS) ordination, followed by permANOVA tests, to compare the composition of visitor orders (bees, flies, and beetles) between repetitions, locations, colours, and monitoring methods (flower arrays versus pan traps). The NMDS analysis included data collected in 2023 only, when both observations from pan traps and flowers were available. Because insects cannot revisit after being caught in a trap, comparing the two monitoring methods is tricky. To overcome this challenge, we normalized our results by only including the first visit of each insect in the flower arrays. This generated a single colour choice record per visitor, whether it landed in a flower array or in a pan trap.

#### Predictors of visit rates to each flower colour

We examined which experimental variables predict visitation to each of the floral colour morphs (red/purple/white) using generalized linear models (GLMs). For example, in the model for red-flower visits, we scored each visit as being to a red flower or not, coding flower colour as a binomial dummy variable. We repeated this procedure for white and for purple flowers. This generated three logistic binomial regression models with log link functions. Each model tested the effects of time in the season (early/mid/late), location (south/centre/north), and visitor type (bee/fly/beetle) on the binary choice of red, white, or purple. We created reduced models by removing explanatory variables one at a time. We then used likelihood ratio tests to compare the full models to the reduced ones and to calculate the statistical significance of each variable.

We used the packages plotrix, ggplot2, lme4, tidyr, dplyr, lmtest, reshape2, vegan, and MASS, implemented in R version 4.2.1 (R Core Team 2022) for the analyses.

## Results

### Insect visits to flower arrays

#### Insects increasingly visited the southern site along the flowering season

**Figure 2:**
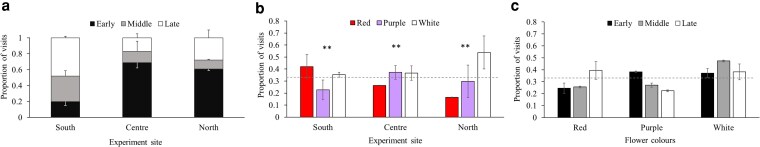
**(a)** Insect visitations in the flower arrays during seasons 2022–2023. The proportions of visits during early, mid, and late season were calculated for each site separately and averaged over the 2 years of the study. Mean values are plotted with the associated standard errors. **(b)** Floral colour preferences of visiting insects in the flower array, years 2022–2023. Visitations are categorized according to colour, along a latitudinal gradient. Proportions were averaged over the 2 years of the study. Means are plotted with the associated standard errors. The proportions of visits to all three colours in each location (south, centre, and north) sum up to 1. Asterisks denote significant deviation from uniform distributions (χ^2^ tests, *P* < 0.01). The dashed line at 1/3 signifies the proportion of insect visits to each colour expected under the null hypothesis of no colour preferences. **(c)** Insect visitations in the flower arrays along seasons 2022–2023. Proportions were averaged over the 2 years of the study. Means are plotted with the associated standard errors. The proportions of the three timings in the season (early, mid, and late) sum up to 1. The dashed line at 1/3 signifies the proportion of insect visits expected in each time-point under the null hypothesis of a uniform distribution.

Insect visits in the central and northern study sites occurred mainly at the beginning of the flowering season (January). In the southern site, on the other hand, most visits were recorded in the experiment’s last repetition, in late season (Fig. 2a, Supplementary Table S2a). The distribution of visits across the three repetitions of the experiment, pooled over the 2 years, differed significantly from uniform in all sites. When tested separately for each year, the distribution of visits differed significantly from uniform in all sites, apart from the centre during year 2022 (Table 2).

#### Red flowers received most insect visits in the south, white in the north

During 2022, red, white, and purple flowers received 101, 185, and 157 visits, respectively, and during the 2023 season they received 425, 531, and 423 visits, respectively. The proportions of visits to the three colours varied over space: the proportion of visits to red flowers declined from the southern site to the northern one, while the proportion of visits to white flowers increased (Fig. 2b, see Supplementary Table S2b for a partitioning of the data by year). The distribution of visits between the colour morphs, pooled over all repetitions, differed from random expectation in all sites (northern site, white preferred: χ^2^_2_ = 69.62; central site, white preferred: χ^2^_2_ = 40.81; southern site, red preferred: χ^2^_2_ = 28.78; *P* < 0.001 for all sites).

#### Red flowers received most visits during late season, purple flowers during early season

Visits to red flowers increased in frequency from early to late season, whereas the frequency of visits to purple flowers decreased (Fig. 2c, see Supplementary Table S2c for the data separated by year). The distribution of visits among flower colours deviated from uniform in all three repetitions (early season, red flowers were under-visited: χ^2^_2_ = 34.24; mid season, white flowers were preferred: χ^2^_2_ = 60.59; late season, purple flowers were under-visited: χ^2^_2_ = 27.39; *P* < 0.001 for all tests).

### Flower arrays and pan-trap captures: visitor composition and colour preferences

#### Coleoptera dominated flower arrays in the south, Diptera and Hymenoptera in the north

**Figure 3:**
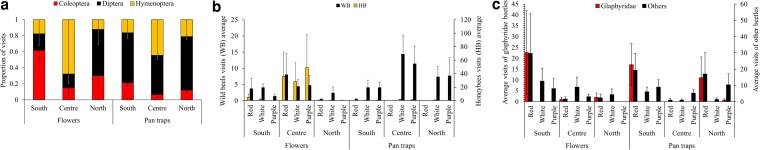
**(a)** Insect composition in the flower arrays and pan traps in the three study sites in 2023. Only the first visit to a flower per insect individual was considered, thus each individual contributes one choice record. The relative abundances of bees, flies, and beetles varied more strongly between sites on the flower arrays than in the pan traps. **(b)** Numbers of bee visitors observed during a day (mean + SE) in the flower arrays (‘Flowers’) and captured in pan traps (‘Pan traps’) along the latitudinal gradient in 2023. The primary and secondary *y*-axes depict wild bees and honeybees, respectively. Note the different *y*-axis scales. **(c)** Mean daily numbers of beetle visitors in the flower arrays (‘Flowers’) and captures in pan traps (‘Pan traps’) along the latitudinal gradient in 2023, glaphyrids versus beetles from all other families. Note the different y-axis scales.

We compared between the composition of insect visitors to flowers and the composition of captures in nearby pan traps to test whether they show similar trends along time and space. Overall, we observed 886 bees, 1242 flies, 614 beetles, and 2648 insects from other orders in both flower arrays and pan traps combined (see Supplementary Table S3). Coleoptera was the dominant insect order in the flower arrays, but not in the pan traps, in the southern site. In the central and northern sites, Diptera and Hymenoptera predominated both in the pan traps and on the flowers (Fig. 3a).

#### Coleoptera dominated flower arrays in late season, Diptera and Hymenoptera in early season

**Figure 4:**
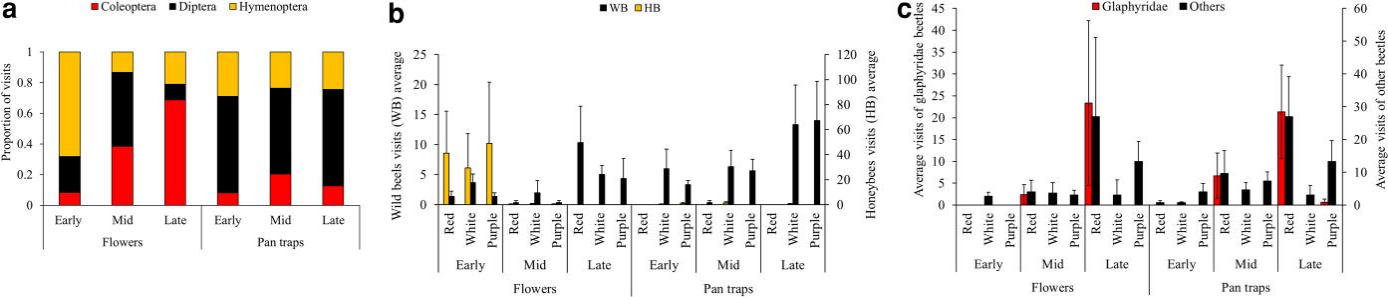
**(a)** Insect visitor composition shifts in the flower arrays and pan traps over time along 2023. Pan trap captures provide a reference to the seasonal variability in insect composition. SEs were not plotted for graphical clarity, and are provided in Supplementary Table S5. **(b)** The composition of honey bees and wild bees in pan traps versus flowers along the 2023 season. Note the differences in *y*-axis scales. **(c)** Beetle visits in pan traps versus flowers along the 2023 season, Glaphyrid beetles versus beetles from all other families. Note the different y-axis scales.

The frequency of Coleoptera as flower visitors increased along the season, but their representation in the pan traps remained low. The frequency of Hymenoptera among flower visitors, on the other hand, declined along the season (Fig. 4a). The combined evidence from the seasonal and geographical trends in beetle observations, and the consistently low Coleoptera captures in pan traps, indicates that the beetles’ attraction to anemones increased towards the southern site and as the season progressed, in line with their association with higher temperatures. Under these conditions, beetles numerically replaced hymenopterans in the flower arrays.

#### Honey bees, glaphyrid beetles, and syrphid flies were dominant visitors

To gain a more detailed picture of the visitors’ colour choices, we categorized hymenopteran visitors into either domesticated honey bees or wild solitary bees (Fig. 3b and 4b). We also separated records of beetles from the family Glaphyridae, previously described as key visitors of red anemones (Keasar *et al*. 2008, Streinzer *et al*. 2019), from all other beetles (Fig. 3c and 4c). Both honey bees and wild bees avoided the red pan traps, but visited red flowers in the flower arrays, especially in the central site (Fig. 3b). Most of the bee visits during the early season were by the honey bees (Fig. 4b), most probably arriving from agricultural areas bordering our study site. Among beetles, the family Glaphyridae dominated the red flowers as well as the red pan traps, particularly in the southern site (Fig. 3c) and during the late part of the flowering season (Fig. 4c). Further identification of the Coleopteran visitors to family level showed that other beetle groups, such as from the family Chrysomalidae, were mostly caught in the southern site in late season but did not specialize on red flowers or red pan traps (see Supplementary Figs. S2, S3; and Tables S5, S6, S7). Diptera were the most common order of visitors, and syrphid flies were particularly dominant visitors within this order, as previously described (Dafni *et al*. 2020). Indeed, we found that syrphids accounted for ∼50% of all visits of flies to purple flowers, and ∼30% visits to white, but nearly none of the visits to red flowers. Other dipteran visitors were not analysed in detail, because their contribution to pollination is not sufficiently known. The visitors’ order-level composition was best explained by the timing in season (permANOVA, *r*^2^ = 0.118, *P* = 0.001), followed by the site location (*r*^2^ = 0.075, *P* = 0.006), flower/pan-trap colour (*r*^2^ = 0.064, *P* = 0.017), and monitoring method (flower or trap) (*r*^2^ = 0.063, *P* = 0.003).

#### Site location and visitor type predict visits to each of the colours

When focusing on each flower colour morph individually (see Supplementary Table S4), the numbers of visits to red and to white flowers were significantly predicted by the site location, visitor type, and time in the season (GLM, *P* < 0.001 for all three factors). For the purple flower colour, visitation rates were significantly influenced by location (GLM, *P* = 0.004) and by visitor type (GLM, *P* < 0.001), but not by the time in the season (*P* = 0.31). Red and purple flower visits were also predicted by year (GLM, *P* = 0.01 and *P* < 0.001, respectively), while white flower visits did not differ among years (GLM, *P* = 0.5).

## Discussion

Within-species FCP occurs more frequently among plant populations than within populations (Sapir *et al.* 2021). Flower colour polymorphism along a plant’s distribution range is often attributed to changes in pollinator composition that select for different flower colours (Streinzer *et al*. 2019, Johnson *et al*. 2025). Several previous studies aimed to explain the variation in flowering colour along a geographical gradient by testing how pollinator communities shift along the gradient (Streisfeld and Kohn 2007, Kemp *et al*. 2019, Koski *et al*. 2020, Wenzell *et al*. 2023, Johnson *et al.* 2025). Studies that relate the seasonal turnover of pollinators as drivers of FCP are scarce (Keasar and Labin 2025). This study advances previous research by simultaneously addressing pollinator-related FCP across spatial (northern, central, and southern sites) and temporal (early, mid, and late season) gradients, and by examining their interaction. This enables us to address the study question regarding the role of pollinators in driving and maintaining FCP through time and space.

We asked how visitor activity and composition vary along the plant’s distribution and over the season, and discovered that most insect visits in the northern and central sites occurred in early season, while the southern site received most visits in late season (Fig. 2a). Next, we asked how the visitors’ flower colour choices vary along the north−south gradient and over the season. We found that bees and flies visited purple and white flowers at higher frequencies than red flowers in the northern and central sites, mostly during the beginning of flowering season, and that Glaphyrid beetles visited red flowers most often in the southern site later in the season (Figs. 2b-2c and 3a and 4a). Table 1 summarizes the key features of the colour vision system in the most common families of insect visitors in our study. It suggests that the visitors’ visual capabilities may have played a role in their colour choices, along with other mechanisms (Table 1; van der Kooi *et al*. 2021). Some of the shifts in visitor composition may reflect their different phenology, as previous studies on *A. coronaria* report early-season emergence of some wild bees, whereas beetles emerge later and increase their activity on warm days (Horovitz 1991, Keasar *et al*. 2008, Keasar *et al.* 2010, Tzohari *et al*. 2012, Dafni *et al*. 2020, Keasar and Labin 2025). We conclude that the observed variation in visitor composition and colour choice is consistent with the spatial and temporal pattern of FCP in *A. coronaria*. Namely, purple and white anemones grow in northern and central sites and flower early, whereas red anemones flower later and are the only colour morph in southern sites (Horovitz *et al*. 1975, Keasar and Labin 2025, Labin *et al*. 2025).

**Table 1:** Key features of the colour vision systems in hymenopteran, dipteran, and coleopteran pollinators.

Pollinator order	Pollinator (genera)	Number of spectral photoreceptor types	Peak colour sensitivity of photoreceptors (reviewed by van der Kooi *et al.* 2021)	Photoreceptor sensitivity to red	References
Hymenoptera	Andrenidae (*Andrena, Callonychium, Oxaea*)	3–4	348–370 435–445 529–536 593	Low	Peitsch *et al*. (1992), Qiu *et al.* (2002), Arikawa (2003), Land and Nilsson (2012)
	Apidae (*Apis, Bombus, Lestrimelitta, Melecta, Melipona, Nomada, Partamona, Proxylocopa, Schwarziana, Trigona, Xylocopa*)	3	329–362 424–453 512–544	Low	
	Halictidae (*Lasioglossum*)	3	Missing data 442 516–528	Low	
	Megachilidae (*Anthidium, Chelostoma, Osmia*)	3	324–356 445 531–553	Low	
Diptera	Syrphidae (*Allograpta, Eristalis, Syrphus, Toxomerus*)	5	350 Missing data 450–480 Missing data 520	Low	Troje (1993), Lunau and Knüttel (1995), Kelber *et al.* (2003), An *et al.* (2018)
Coleoptera	Chrysomelidae (*Leptinotarsa*)	3	370/ND 420 520/ND	Low	Booth *et al.* (2004), Doering *et al.* (2012), Martínez-Harms *et al*. (2012), Sharkey *et al.* (2017), Belušič *et al.* (2025)
	Glaphyridae (*Pygopleurus*)	4	360 430 517 631	High	
	Scarabaeidae (*Anomala, Lethrus, Onitis, Protaetia*)	3	355–400 460 498–562	Low	
	Other families	2–5	UV, green and purple-blue in several taxa, unknown in many others	Low in most studied taxa, high in Carabidae and Glaphyridae	

The photoreceptors in the peak colour sensitivity column are ordered from short to long—a single row for each receptor. ND = not determined.

**Table 2:** The outputs of χ^2^ tests, which evaluated whether insect visits were uniformly distributed between the early-season, mid-season, and late-season repetitions of the experiment.

Years	2022 and 2023		2022		2023	
Category	χ^2^_2_	*P*	χ^2^_2_	*P*	χ^2^_2_	*P*
South	60.37	<0.001	30.45	<0.001	43.366	<0.001
Centre	568.03	<0.001	4.03	0.133	671.42	<0.001
North	126.80	<0.001	114.54	<0.001	39.45	<0.001

We conducted the tests with pooled data from the 2 years of the study, as well as with each year’s data separately.

Along our study, we documented diverse insect visitors to *A. coronaria*. The plant’s broad geographical distribution and extended flowering period (Horovitz *et al.* 1975) may expose it to seasonal changes over time and to variable abiotic characteristics over space (Dafni *et al*. 2020, Labin *et al*. 2025), linked to multiple pollinator assemblages that vary in their colour preferences. We propose that FCP in *A. coronaria* may support the plants’ generalized interactions with insect pollinators. In addition, *A. coronaria* does not rely solely on insect pollination, but also combines wind pollination, further enhancing its generalized pollination strategy (Dafni *et al*. 2020).

The abundance of co-flowering species in the plant community is an additional potential element of the change in visitor composition in our *A. coronaria* flower arrays (Seifan *et al*. 2014). *A. coronaria* starts flowering in early winter, when there are few co-flowering species. Later during spring flower abundance and competition for pollinators increase (Menzel and Shmida 1993). We conjecture that honey bees, wild bees, and flies may have responded to the increased flower abundance by reducing visits to *A. coronaria* (Fig. 2a). Empirical testing of this possibility requires pollinator observations in the co-flowering plant communities along *A. coronaria*’s flowering season, and exceeds the scope of the present study.

We also found that proportions of visits to red and purple flowers varied between the 2 years of the study, while visits to white flowers remained stable. We speculate that these shifts might be linked to the average temperature along the flowering season, which was higher in the second season than in the first one (average temperatures during our measurement days: year 2022–15.66°C; year 2023–16.58°C). Higher average seasonal temperatures could favour the insect visitors that are attracted to red flowers. Colder winters may benefit the purple morph by extending the conditions of the early season, the period in which it receives most visits. Experiments under temperature-controlled conditions are needed to evaluate these speculations.

Most past studies on *A. coronaria* and its reproduction and pollination services focused on glaphyrids. In these studies, Glaphyridae were strictly associated with red flowers (Keasar *et al*. 2008, Keasar *et al*. 2010, Dafni *et al*. 2020), a pattern explained by their spectral sensitivity extending into the red range and their strong preference for red stimuli (Belušič *et al*. 2025). Here, we expanded our examination to other beetle families, and found that they have more diverse flower preferences. We observed that dominant beetle visitors of the families Chrysomelidae and Ripiphoridae preferentially fed on the pollen of non-red flowers, mostly in late season. Additionally, Oxythyrea (Scarabaeidae) beetles showed particular preference to white flowers. We suspect that these visitors provide complementary pollination to non-red flowers in the Mediterranean area, especially during late season, when visits by bees and flies decline. Further studies are needed to quantify the pollination service provided by non-glaphyrid beetles, such as a bruchinid species that was the most common visitor within the family Chrysomelidae (see Supplementary Figure S2, S3). In addition, the pollination efficacy of each group of floral visitors should be evaluated (Heinze *et al*. 2026), to quantify their relative contribution to pollination (Ne’eman *et al*. 2010). Interestingly, although bees preferred non-red flowers in the Mediterranean area, they opted for red flowers in the south. Bees are known for their effective colour learning capabilities linked with foraging behaviour (Hempel de Ibarra *et al*. 2014). Since red is the only anemone floral colour naturally occurring in the south, we hypothesize that the bees’ preference of red involved learning. Apart from the red preference of bees in the south, the colour choices of the three insect orders aligned with their visual capabilities and sensitivities (Fig. 1, see Supplementary Figure S1, Table 1).

FCP has evolved independently in multiple clades within the genus *Anemone (e.g. A. acutiloba*, *A. americana*, *A. nemorosa*, *A. trullifolia*, *A. pavonina*, *A. palmata*, and *A. obtusiloba*; Hoot *et al*. 2012). Interestingly, these species share a suite of ecological characteristics with *A. coronaria*, including all or most of the following: (i) an extended flowering season, (ii) a broad distribution range, (iii) early blooming, and (iv) generalized pollination interactions (Bernhardt 1976, Horovitz 1976, Motten 1982, Horovitz 1991, Murphy and Vasseur 1995, Laura *et al*. 2006, Meng *et al.* 2008, Keasar *et al*. 2010, Warren and Bradford 2014, Liu *et al*. 2017, Hu and Wu 2019, Streinzer *et al*. 2019, Dafni *et al*. 2020, Hu *et al*. 2020, Sevenello *et al*. 2020, Ding *et al.* 2022, Puchałka *et al.* 2022, Rodríguez-Castañeda *et al*. 2024, Heinze *et al*. 2026). Previous studies proposed potential selective benefits for these traits, such as improved reproductive assurance provided by the long flowering season (Tzohari *et al*. 2012) and reduced competition for pollinators achieved by early flowering (Bernhardt 1976, Morten 1982). At the same time, the need to attract diverse pollinators may present a challenge for broadly distributed species with long flowering seasons. FCP may alleviate this challenge in *A. coronaria* as well as in its colour-polymorphic congeners because multiple colour morphs can attract a wide range of pollinators (Bernhardt 1976, Motten 1982, Horovitz 1991, Bartomeus *et al*. 2013, Dafni *et al*. 2020, Sevenello *et al*. 2020). FCP may also provide additional environmental adaptations, based on flavonoid pigments that offer protection from abiotic stressors such as radiation, aridity, or cold (Gould 2004, Landi *et al*. 2015, Labin *et al*. 2025).

We therefore propose that a suite of life-history traits drives generalized pollination interactions and selects for the coexistence of colour morphs in several *Anemone* species. Species that cover a broad distribution range, possess a long flowering season, and have selfing barriers, may benefit from diverse pollination strategies. These strategies include combining insect and wind pollination and maintaining polymorphic floral displays that attract diverse insect pollinators. Temporal partitioning in flowering phenology of different colours, as known in several *Anemone* species, may increase the chances of fertilization between individuals of the same colour while limiting random cross-colour fertilization, hence maintaining those colour traits. Overall, we conclude that the variety of biotic and abiotic factors the plant encounters generate diverse ecological niches in time and space, which altogether may support FCP as part of a generalist reproductive strategy. Our findings shed light on the role of pollinators in maintaining diverse floral colours in *A. coronaria.* Non-pollinator biotic and abiotic factors intricately affect the fitness of *A. coronaria*’s colour morphs (Labin *et al*. 2025, Saabna *et al*. 2025), and potentially interact with pollinators in driving this FCP.

## Supplementary Material

plag009_Supplementary_Data

## Data Availability

Raw data and R code are available online in Zenodo, at https://zenodo.org/records/17347179
