# Supplementary material for: Flower colour polymorphism in Anemone coronaria correlates with the activity pattern and colour preferences of its visitors
Source: AoB Plants. 2026 Feb 18;18(2):plag009. doi: 10.1093/aobpla/plag009 (PMC12952293; doi:10.1093/aobpla/plag009)
Supplement: plag009_Supplementary_Data [file plag009_supplementary_data.zip › Supporting Information Table S1.fixed.docx]

**Supporting Information Table S1**

**Table S1**. Abiotic conditions recorded on the day flower-visitor observations were taken. The column ‘Rain’ describes whether it rained on the day prior to the experiment. The column ‘Cloud coverage’ describes the coverage of the sky in intervals of 1/8, between 0-1.

| Date | Site | Rain | Shade temp. | Sun temp. | Wind (strong/medium/light) | Cloud coverage |
| --- | --- | --- | --- | --- | --- | --- |
| 16/02/2022 | South | No | 12.9 | 13.4 | Medium | 0.875 |
| 17/02/2022 | Centre | No | 10.3 | 11.2 | Light | 0.125 |
| 18/02/2022 | North | No | 12.6 | 13.7 | Light | 0 |
| 07/03/2022 | North | NAN | 15.6 | 16.6 | Light | 0 |
| 09/03/2022 | South | No | 20.6 | 22.2 | Light | 0.25 |
| 27/03/2022 | Centre | No | 14.9 | 15.8 | Light | 0.5 |
| 28/03/2022 | South | No | 13 | 14.9 | Light | 0 |
| 29/03/2022 | Centre | No | 18.5 | 20.1 | Medium | 0.125 |
| 30/03/2022 | North | No | 17.3 | 18.4 | Light | 0 |
| 18/01/2023 | North | No | 13.3 | 13.6 | Light | 0 |
| 22/01/2023 | Centre | No | 12.5 | 14.1 | Light | 0.125 |
| 28/01/2023 | South | No | 20 | 20.7 | Strong | 0.875 |
| 19/02/2023 | South | No | 15 | 16.7 | Light | 0 |
| 22/02/2023 | North | No | 12 | 16.1 | Light | 0 |
| 23/02/2023 | Centre | No | 13.5 | 13.3 | Light | 1 |
| 07/03/2023 | Centre | No | 17.1 | 19.7 | Light | 0.875 |
| 12/03/2023 | South | No | 20.3 | 20.8 | Medium | 0 |
| 16/03/2023 | North | Yes | 18.5 | 21.3 | Light | 0.375 |
