# Supplementary material for: Flower colour polymorphism in Anemone coronaria correlates with the activity pattern and colour preferences of its visitors
Source: AoB Plants. 2026 Feb 18;18(2):plag009. doi: 10.1093/aobpla/plag009 (PMC12952293; doi:10.1093/aobpla/plag009)
Supplement: plag009_Supplementary_Data [file plag009_supplementary_data.zip › Supporting Information Table S2 (a, b, c).fixed.docx]

**Supporting Information Table S2 (a, b, c)**

**Table S2) a(** Insect visitations in the flower arrays during seasons 2022-2023, as shown in Fig. 2a. The data are separated by year and are also averaged over the two years of the experiment. The ‘Counts’ columns list the numbers of visits, and the ‘Proportions’ columns present the proportion of visits along the season in each experimental site. The SE column reports the variation between the two years of the study.

|  |  | Counts | | | Proportions | | | |
| --- | --- | --- | --- | --- | --- | --- | --- | --- |
| Site location | Time | 2022 | 2023 | Average 2022,2023 | 2022 | 2023 | Average 2022, 2023 | SE (prop. 2022,2023) |
| South | Early | 15 | 86 | 50.5 | 0.110 | 0.233 | 0.200 | 0.050 |
|  | Mid | 60 | 101 | 80.5 | 0.441 | 0.274 | 0.319 | 0.068 |
|  | Late | 61 | 182 | 121.5 | 0.449 | 0.493 | 0.481 | 0.018 |
| Centre | Early | 41 | 641 | 341 | 0.308 | 0.750 | 0.180 | 0.690 |
|  | Mid | 55 | 82 | 68.5 | 0.414 | 0.096 | 0.130 | 0.139 |
|  | Late | 37 | 132 | 84.5 | 0.278 | 0.154 | 0.051 | 0.171 |
| North | Early | 126 | 77 | 101.5 | 0.712 | 0.490 | 0.090 | 0.608 |
|  | Mid | 22 | 16 | 19 | 0.124 | 0.102 | 0.009 | 0.114 |
|  | Late | 29 | 64 | 46.5 | 0.164 | 0.408 | 0.100 | 0.278 |

**Table S2) b(** Floral colour preferences of visiting insects in the flower array, years 2022-2023, as shown in Fig. 2b. The data are separated by year and are also averaged over the two years of the experiment. The ‘Counts’ columns list the numbers of visits, and the ‘Proportions’ columns present the proportion of visits to each flower colour in each experimental site. The SE column reports the variation between the two years of the study.

|  |  | Counts | | | Proportions | | | |
| --- | --- | --- | --- | --- | --- | --- | --- | --- |
| Site location | Time | 2022 | 2023 | Average 2022,2023 | 2022 | 2023 | Average 2022, 2023 | SE (prop. 2022,2023) |
| South | Red | 37 | 175 | 106 | 0.272 | 0.474 | 0.420 | 0.101 |
|  | Purple | 47 | 68 | 57.5 | 0.346 | 0.184 | 0.228 | 0.081 |
|  | White | 52 | 126 | 89 | 0.382 | 0.341 | 0.352 | 0.020 |
| Center | Red | 34 | 225 | 129.5 | 0.258 | 0.263 | 0.262 | 0.003 |
|  | Purple | 36 | 331 | 183.5 | 0.273 | 0.387 | 0.372 | 0.057 |
|  | White | 62 | 299 | 180.5 | 0.470 | 0.350 | 0.366 | 0.060 |
| North | Red | 29 | 25 | 27 | 0.167 | 0.161 | 0.164 | 0.003 |
|  | Purple | 74 | 24 | 49 | 0.425 | 0.155 | 0.298 | 0.135 |
|  | White | 71 | 106 | 88.5 | 0.408 | 0.684 | 0.538 | 0.138 |

**Table S2) c(** Insect visitations in the flower arrays along seasons 2022-2023, as shown in Fig. 2c. The data are separated by year and are also averaged over the two years of the experiment. The ‘Counts’ columns list the numbers of visits, and the ‘Proportions’ columns present the proportion of visits to each flower colour in each repetition of the experiment along the season. The SE column reports the variation between the two years of the study

|  |  | Counts | | | Proportions | | | |
| --- | --- | --- | --- | --- | --- | --- | --- | --- |
| Flower colour | Time | 2022 | 2023 | Average 2022,2023 | 2022 | 2023 | Average 2022, 2023 | SE (prop. 2022,2023) |
| Red | Early | 32 | 210 | 121 | 0.176 | 0.262 | 0.246 | 0.043 |
|  | Mid | 33 | 52 | 42.5 | 0.246 | 0.263 | 0.256 | 0.008 |
|  | Late | 36 | 163 | 99.5 | 0.283 | 0.431 | 0.394 | 0.074 |
| White | Early | 69 | 297 | 183 | 0.379 | 0.370 | 0.372 | 0.038 |
|  | Mid | 66 | 91 | 78.5 | 0.493 | 0.460 | 0.473 | 0.008 |
|  | Late | 50 | 143 | 96.5 | 0.394 | 0.378 | 0.382 | 0.066 |
| Purple | Early | 81 | 296 | 188.5 | 0.445 | 0.369 | 0.383 | 0.005 |
|  | Mid | 35 | 55 | 45 | 0.261 | 0.278 | 0.271 | 0.016 |
|  | Late | 41 | 72 | 56.5 | 0.323 | 0.190 | 0.224 | 0.008 |
