# Supplementary material for: Flower colour polymorphism in Anemone coronaria correlates with the activity pattern and colour preferences of its visitors
Source: AoB Plants. 2026 Feb 18;18(2):plag009. doi: 10.1093/aobpla/plag009 (PMC12952293; doi:10.1093/aobpla/plag009)
Supplement: plag009_Supplementary_Data [file plag009_supplementary_data.zip › Supporting Information Table S3.fixed.docx]

**Supporting Information Table S3**

**Table S3**. Numbers of visitors to flower arrays and pan traps during the study.

|  | Bees | Flies | Beetles | Other orders |
| --- | --- | --- | --- | --- |
| Flower arrays | 506 | 297 | 403 | 270 |
| Pan traps | 380 | 945 | 211 | 2378 |
