# Supplementary material for: Flower colour polymorphism in Anemone coronaria correlates with the activity pattern and colour preferences of its visitors
Source: AoB Plants. 2026 Feb 18;18(2):plag009. doi: 10.1093/aobpla/plag009 (PMC12952293; doi:10.1093/aobpla/plag009)
Supplement: plag009_Supplementary_Data [file plag009_supplementary_data.zip › Supporting Information Table S4.docx]

**Supporting Information Table S4**

**Table S4**. Summaries of GLM models of the factors that influenced insect visits to each floral morph.

|  | Effect of | | | | | | | | | | | |
| --- | --- | --- | --- | --- | --- | --- | --- | --- | --- | --- | --- | --- |
| Category / Flower colour | Site | | | Visitor type | | | Time in season | | | Year | | |
|  | χ^2^ | DF | *P* | χ^2^ | DF | *p* | χ^2^ | DF | *p* | χ^2^ | DF | *p* |
| Red | 43.748 | 2 | 3.164e-10 | 89.608 | 3 | < 2.2e-16 | 49.045 | 2 | 2.239e-11 | 6.327 | 1 | 0.012 |
| Purple | 10.843 | 2 | 0.004 | 36.389 | 3 | 6.196e-8 | 2.3187 | 2 | 0.314 | 12.595 | 1 | 0.3e-3 |
| White | 34.362 | 2 | 3.454e-08 | 153.74 | 3 | < 2.2e-16 | 24.602 | 2 | 4.548e-06 | 0.3815 | 1 | 0.537 |
