# Supplementary material for: Flower colour polymorphism in Anemone coronaria correlates with the activity pattern and colour preferences of its visitors
Source: AoB Plants. 2026 Feb 18;18(2):plag009. doi: 10.1093/aobpla/plag009 (PMC12952293; doi:10.1093/aobpla/plag009)
Supplement: plag009_Supplementary_Data [file plag009_supplementary_data.zip › Supporting Information Table S5.fixed.docx]

**Supporting Information Table S5**

**Table S5**. SEs of insect-visitors’ proportions in the flower arrays and pan traps along 2023.

|  |  | Coleoptera | Diptera | Hymenoptera |
| --- | --- | --- | --- | --- |
| Flower arrays | Early | 0.113941 | 0.313962 | 0.385694 |
|  | Mid | 0.540059 | 0.270435 | 0.203994 |
|  | Late | 0.479564 | 0.028985 | 0.136596 |
| Pan traps | Early | 0 | 0.422709 | 0.221821 |
|  | Mid | 0.290164 | 0.333515 | 0.420762 |
|  | Late | 0.191481 | 0.481065 | 0.282269 |
