# Supplementary figures and images for: Flower colour polymorphism in Anemone coronaria correlates with the activity pattern and colour preferences of its visitors
Source: AoB Plants. 2026 Feb 18;18(2):plag009. doi: 10.1093/aobpla/plag009 (PMC12952293; doi:10.1093/aobpla/plag009)

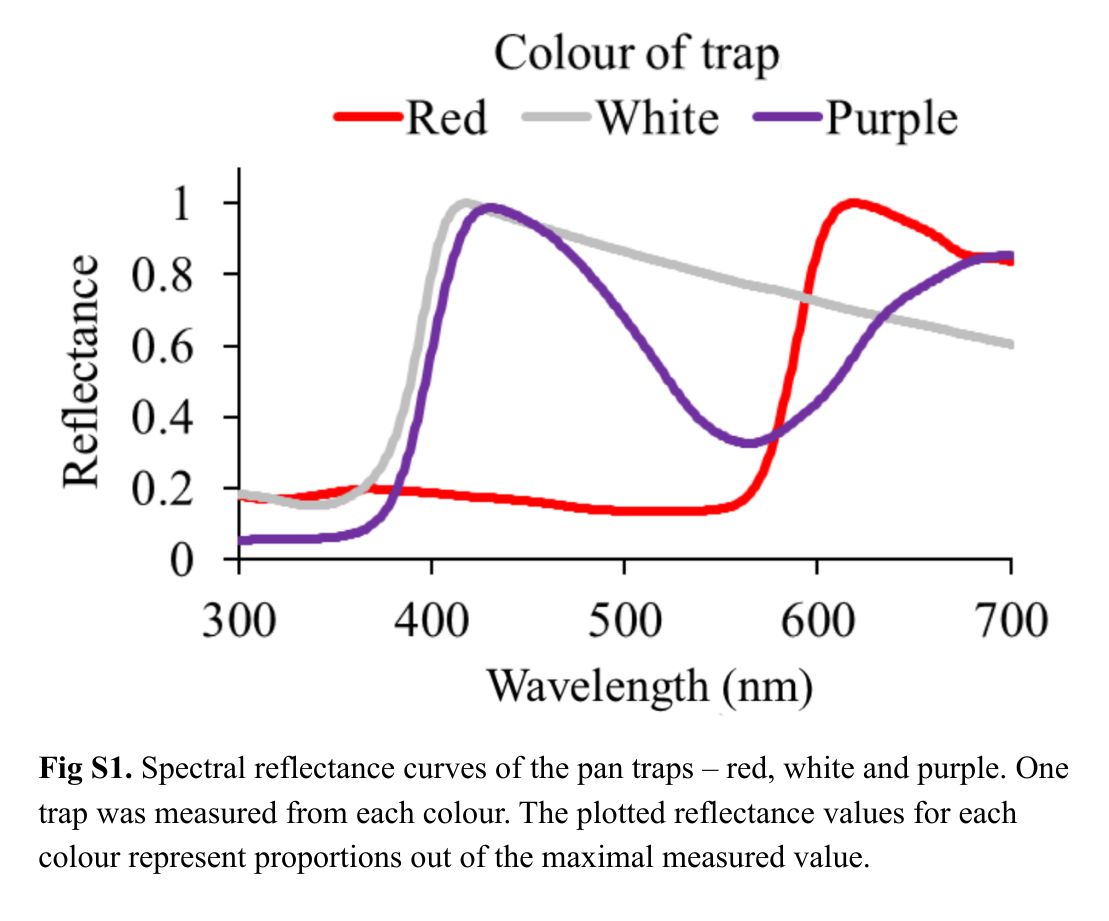

Supplement: plag009_Supplementary_Data [file plag009_supplementary_data.zip › Fig S1.png]

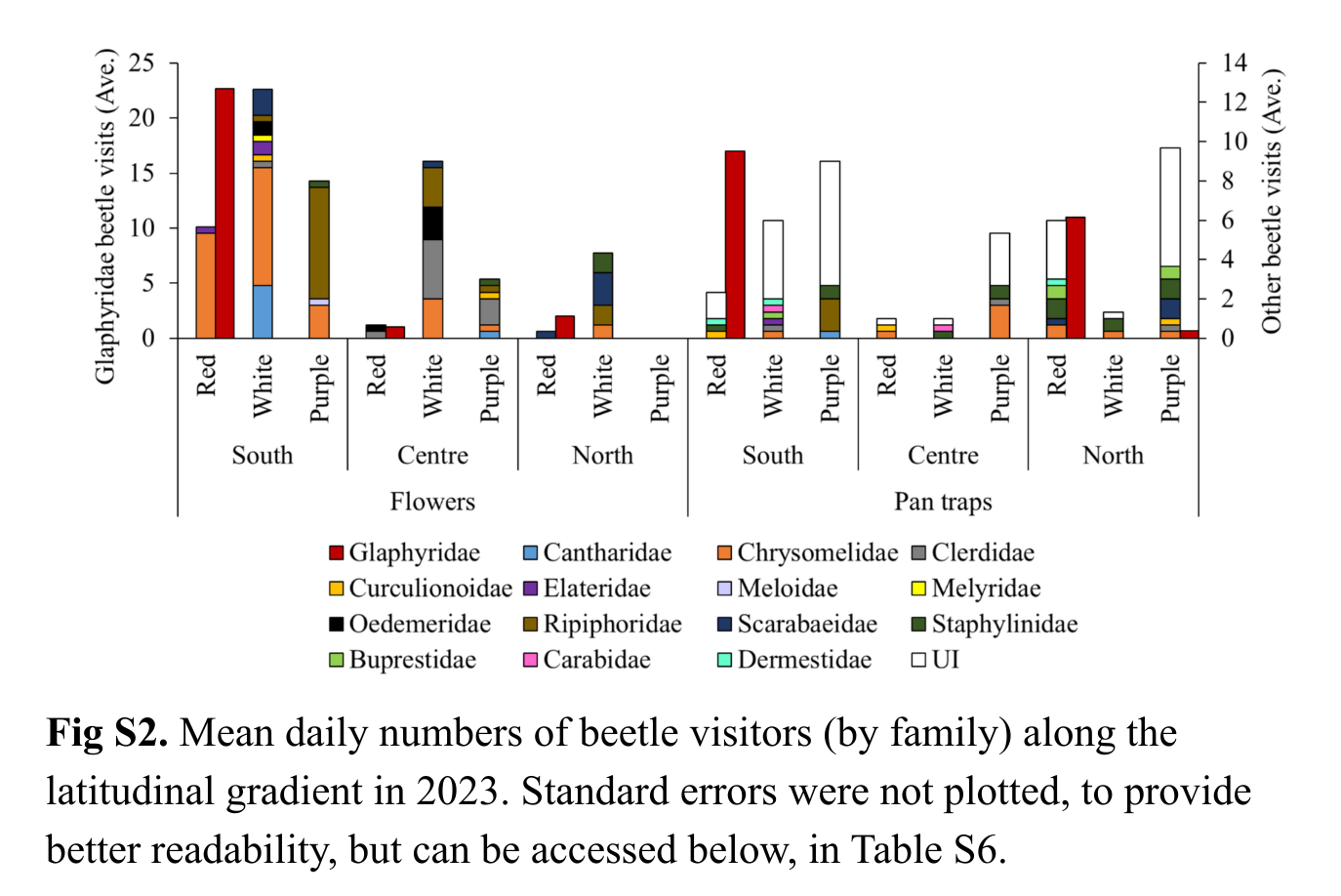

Supplement: plag009_Supplementary_Data [file plag009_supplementary_data.zip › Fig S2..png]

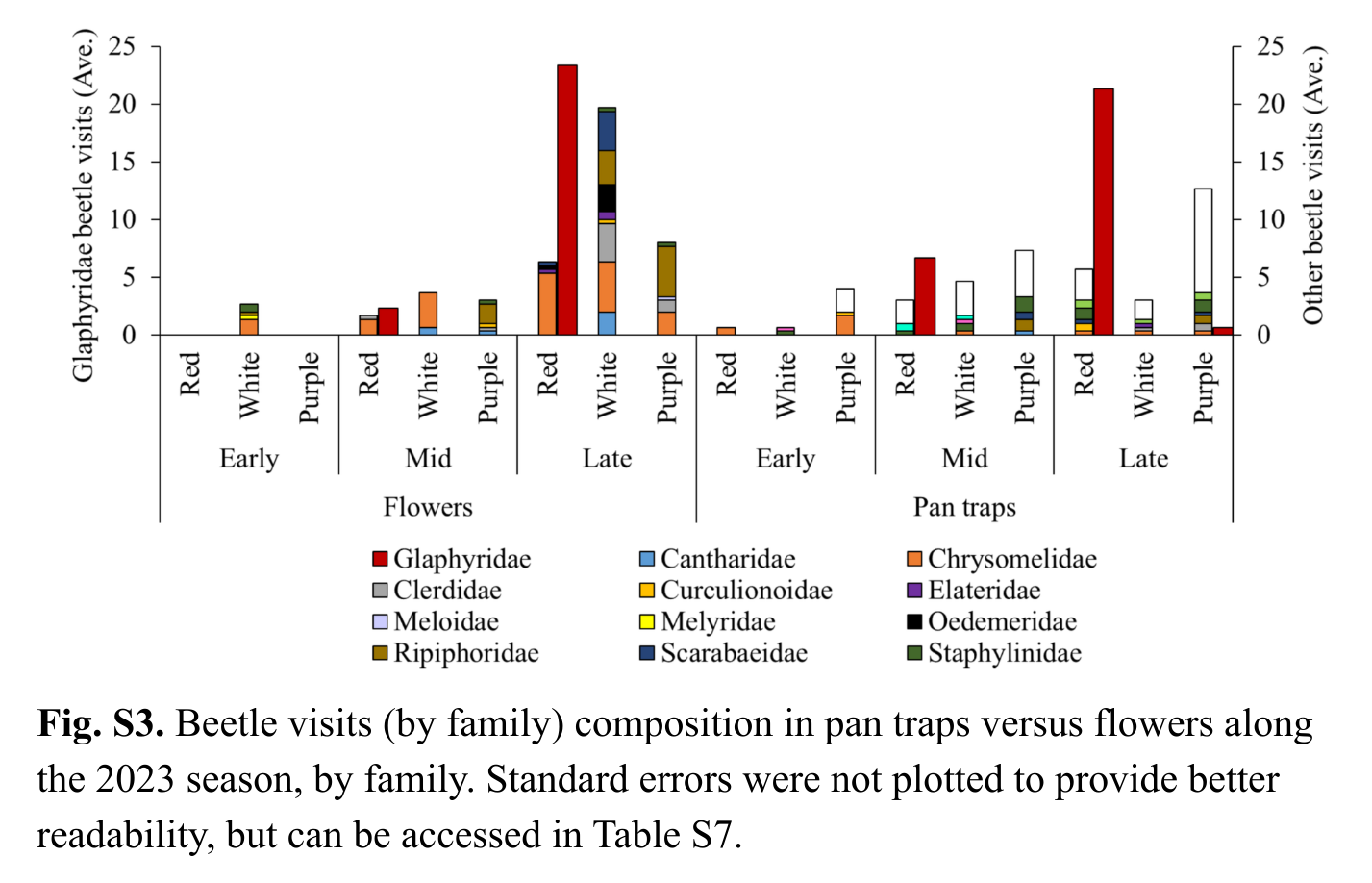

Supplement: plag009_Supplementary_Data [file plag009_supplementary_data.zip › Fig. S3.png]
